# *Candidatus* Coxiella massiliensis Infection

**DOI:** 10.3201/eid2202.150106

**Published:** 2016-02

**Authors:** Emmanouil Angelakis, Oleg Mediannikov, Sarah-Lyne Jos, Jean-Michel Berenger, Philippe Parola, Didier Raoult

**Affiliations:** Aix Marseille Université, Marseille, France

**Keywords:** *Coxiella-*like bacteria, *Candidatus* Coxiella massiliensis, skin biopsy, scalp eschar and neck lymphadenopathy after tick bite, SENLAT, eschar, ticks, bacteria, vector-borne infections, vectorborne, parasites

## Abstract

Bacteria genetically related to *Coxiella burnetii* have been found in ticks. Using molecular techniques, we detected *Coxiella*-like bacteria, here named *Candidatus* Coxiella massiliensis, in skin biopsy samples and ticks removed from patients with an eschar. This organism may be a common agent of scalp eschar and neck lymphadenopathy after tick bite.

Only 1 species of bacteria in the genus *Coxiella* is officially recognized: *Coxiella burnetii* (*1*). However, in recent decades, many genetically related bacteria have been found in hard and soft ticks (*2*). These *Coxiella*-like bacteria genetically cluster with *C. burnetii* and share some degree of identity with these bacteria but not enough to be considered the same species (*2*,*3*). Genotypes of *Coxiella*-like bacteria vary among ticks of different species (*3*); however, bacteria with different genotypes have not been isolated, and whether there is a tick reservoir is not known. *Coxiella*-like bacteria have been associated with infection in birds (*4*,*5*). To explore pathogenicity to humans, we used molecular techniques targeting *Coxiella-*like bacteria to retrospectively analyze skin biopsy samples and ticks collected from patients with eschars. We also evaluated serologic tests for *Candidatus* Coxiella massiliensis diagnosis.

## The Study

During 2011–2014, we identified patients in hospitals throughout France, who had eschars after tick bite. We retrospectively tested skin biopsy or swab samples of the eschars, serum samples when possible, and ticks from the patients. Ticks were identified by matrix-assisted laser desorption/ionization time-of-flight mass spectrometry (Bruker Daltonics, Billerica, USA) (*6*). All human samples and ticks were tested for *Rickettsia*, *Borrelia*, *Bartonella*, and *Ehrlichia* spp.; *Francisella tularensis*; *Staphylococcus aureus*; and *Coxiella burnetii* by quantitative PCR (qPCR) (*2*,*7*). On the basis of the aligned *rrs* gene sequences of *Coxiella*-like bacteria, we developed a specific qPCR to detect the DNA of all *Coxiella* species and degenerated primers aimed to amplify a 659-bps long portion of the *groEL* gene of *Coxiella* spp. (Technical Appendix Table 1). Skin biopsy samples were also tested by universal eubacteria 16S rRNA gene amplification and sequencing (*7*).

Five spawns of *Rhipicephalus sanguineus* ticks infected with *Candidatus* C. massiliensis were used for antigen production (*8*). Molecular assays indicated that these spawns were negative for *Rickettsia*, *Borrelia*, *Bartonella*, *Ehrlichia* spp.; *F. tularensis*; *S. aureus*; and *C. burnetii*. We used spawns of *R. sanguineus* ticks without *Candidatus* C. massiliensis infection as negative controls to confirm that their antigens did not react with serum from the patients. To confirm the presence of *Candidatus* C. massiliensis, we used qPCR and transmission electron microscopy to visualize the bacteria (Technical Appendix Figure). To determine the specificity of our immunofluorescence assay (IFA), we used healthy blood donors as negative controls; to determine if there was cross-reactivity with *C. burnetii*, we used serum from patients with Q fever. 

Patients were considered infected with *Coxiella*-like bacteria when a skin biopsy sample was positive by qPCR and there was no evidence of infection with another agent. Patients were considered possibly infected if they had clinical signs (fever, skin eschar, local lymph node enlargement) and if a removed tick was positive for *Coxiella*-like bacteria according to qPCR but no skin biopsy was sampled or when serologic results were positive. For data comparison, we used Epi Info 6.0 (https://wwwn.cdc.gov/epiinfo/html/ei6_downloads.htm). We considered a p value of 0.05 to be significant.

**Figure 1:**
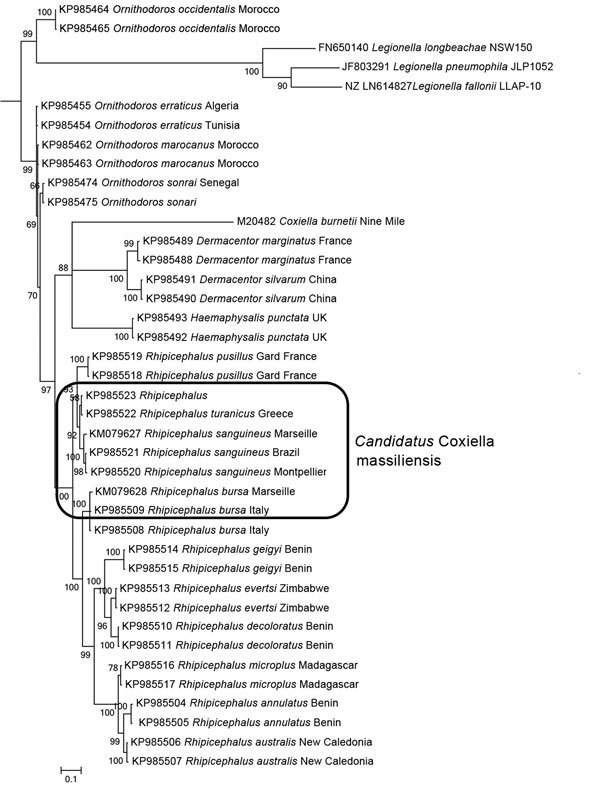
Phylogenetic tree based on *GroEL* sequences including *Coxiella*-like strains of bacteria from ticks, *Coxiella burnetii* reference strains, and bacterial outgroups. *GroEL* gene sequences (Technical Appendix Table 2) were aligned by using ClustalW (http://www.ebi.ac.uk/Tools/msa/), and phylogenetic inferences were obtained by using Bayesian phylogenetic analysis with TOPALi 2.5 software (http://www.topali.org/) and the integrated MrBayes (http://mrbayes.sourceforge.net/) application with the HKY+Г (Hasegawa-Kishino-Yano plus gamma) substitution model for the first and third codons and the JC model for the second codon. GenBank accession numbers are indicated first, followed by the tick host. Numbers at nodes are bootstrap values obtained by repeating the analysis 100 times to generate a majority consensus tree. The final dataset contained 576 positions. Scale bar indicates 10% nucleotide sequence divergence.

Phylogenetic analysis based on *groEL* confirmed that *Coxiella* spp. from ticks of different species are genetically very distant (*9*). On the basis of phylogenetic clustering, epidemiologic role, and the fact that we used its antigens for the diagnostic of human infection, we propose the name for the *Coxiella*-like bacteria associated with *R. sanguineus*, *R. turanicus*, and *H. pusillus* ticks to be *Candidatus* C. massiliensis (Figure 1). 

A total of 57 ticks removed from 55 patients were available for testing. Of these, 20 (35%) ticks from 19 patients were infected with *Coxiella*-like bacteria only: 11 (55%) *Dermacentor marginatus*, 7 (35%) *R. sanguineus*, 1 (5%) *R. bursa*, and 1 (5%) *Ixodes ricinus* ticks. *Coxiella-*like bacteria were found significantly less commonly in *I. ricinus* ticks (p = 0.002, relative risk = 0.5).

**Figure 2:**
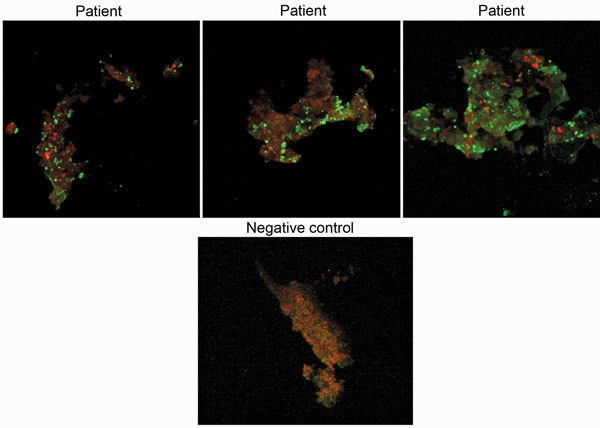
Immunofluorescence assay results of samples from 3 *Candidatus* Coxiella massiliensis–infected patients and 1 noninfected person (negative control). Original magnification ×63.

We tested convalescent-phase serum from 5 patients. Total immunoglobulin titers of 1:400 against *Candidatus* C. massiliensis were detected for 1 patient and >1:800 for 2 patients (Figure 2). All IgG titers obtained were identical. These results indicated an infection caused by *Candidatus* C. massiliensis. IFA results indicated that all patients were negative for *Rickettsia* spp., *C. burnetii*, *F. tularensis*, and *Bartonella* spp. Among serum samples from 40 blood donors, total immunoglobulins titer was 1:200 for 6 donors and IgG titer was 1:400 for 1. Receiver operating characteristic curves, defined by the true-positive rate (serum from patients infected with *Candidatus* C. massiliensis) as a function of the false-positive rate (serum from blood donors) demonstrated that for a total immunoglobulin cutoff of >1:400, sensitivity was 60% and specificity was 100%, and for an IgG cutoff of >1:400, sensitivity was 60% and specificity was 98%. Moreover, among 13 patients with acute Q fever, 12 with Q fever endocarditis, and 5 who had had Q fever in the past, serum was positive for *Candidatus* C. massiliensis, indicating the cross-reactivity of our IFA with *C. burnetii*.

Of 465 skin biopsy samples from 465 patients, cell culture results (*10*) were negative for all. However, samples from 8 (2%) patients were positive for *Candidatus* C. massiliensis, and a possible infection was considered for another 7. Of these 15 patients, 8 (53%) were female, 8 had recently participated in outdoor activities in France, and 2 had recently traveled to Algeria and Israel. Three cases occurred during winter, 9 during spring, and 4 during summer. All patients had an eschar, regardless whether they had lymphadenopathy (Table). A scalp eschar with cervical lymphadenopathy was common (40%). Other common findings were fever (40%), increased C-reactive protein (60%), and thrombocytopenia (40%). Most patients received oral doxycycline, 2 with macrolides and 2 with a β-lactam. Symptoms resolved for all patients.

**Table:** Characteristics of *Candidatus* C. massiliensis–infected patients, France, 2011–2014

Characteristic	No. (%) cases
Male sex	7 (47)
Tick species collected from patient	
* Dermacentor marginatus*	6 (55)
* Rhipicephalus sanguineus*	3 (27)
* Ixodes ricinus*	1 (9)
* Rhipicephalus bursa*	1 (9)
Not collected	4
Eschar location	
Scalp	8 (53)
Leg	3 (20)
Shoulder	2 (13)
Back	1 (7)
Ear	1 (7)
Fever	6 (40)
Lymphadenopathy	
Cervical	6 (55)
Axillary	1 (9)
Inguinal	2 (18)
Thrombocytopenia	6 (40)
Increased liver enzyme levels	2 (13)
Increased C-reactive protein level	9 (60)
Treatment	
Doxycycline	11 (73)
Oher	4 (27)

## Conclusions 

We determined that *Candidatus* C. massiliensis is an etiologic agent of human infections. For our molecular assay, we routinely included large numbers of negative controls that were processed identically to the test samples. Only *Coxiella*-like bacteria DNA was present in the samples, indicating that these bacteria were the only infectious agents. Supplementary serologic testing was used to confirm the molecular results, and results were validated with samples from blood donors; specificity was good but sensitivity was low. The fact that all serum tested demonstrated IgG only does not eliminate the possibility of previous exposure (patients and blood donors) because these bacteria are common in ticks (*9*). Although *Candidatus* C. massiliensis IFA results were cross-reactive with *C. burnetii*, all patients were negative for *C. burnetii*. 

A study limitation was that our assays were specific for *Candidatus* C. massiliensis only. In addition, many of the ticks that bit the patients were not available for examination, and the level of serologic cross-reactivity among *Candidatus* C. massiliensis and other *Coxiella*-like bacteria is unknown. Thus, the patients reported here may have been infected not exclusively with *Candidatus* C. massiliensis but also with another *Coxiella* species associated with ticks.

Most patients had a scalp eschar and cervical lymphadenopathy, reminiscent of a recently proposed clinical entity named SENLAT (scalp eschar and neck lymphadenopathy after tick bite) (*11*). All symptoms arising from *Candidatus* C. massiliensis infection can be easily attributed to other infectious agents transmitted by ticks (*12*,*13*) and can easily go unrecognized because of the absence of systematic research on these bacteria and the nonspecific clinical manifestations of diseases caused by them. Most patients in our study were empirically prescribed doxycycline, the treatment of choice for many tick-transmitted infections (*14*). We illustrated the value of systematically testing for *Candidatus* C. massiliensis in skin biopsy samples. *Candidatus* C. massiliensis may be a common agent of SENLAT, which may go unrecognized or misdiagnosed as a sign of another tick-transmitted infection.

**Online Technical Appendix.** Phylogentic tree of *Coxiella* spp., immunofluorescence assays of persons with and without *Candidatus* Coxiella massiliensis infection, and transmission electron micrograph of *Candidatus* C. massiliensis.
