# Supplementary material for: Candidatus Coxiella massiliensis Infection
Source: Emerg Infect Dis. 2016 Feb;22(2):285–8. doi: 10.3201/eid2202.150106 (PMC4734529; doi:10.3201/eid2202.150106)
Supplement: Supplementary file 1 — Online Technical Appendix. Phylogentic tree of Coxiella spp., immunofluorescence assays of persons with and without Candidatus Coxiella massiliensis infection, and transmission electron micrograph of Candidatus C. massiliensis. [file 15-0106-Techapp-s1.pdf]

Article DOI: http://dx.doi.org/10.3201/eid2202.150106

Candidatus Coxiella massiliensis Infection

Technical Appendix

Technical Appendix Table 1. Primer and probes sequences used for the detection of *Coxiella*- like bacteria

Primer and probes sequences	Targeted gene	Utilization
Forward: ACCTACCCTTGACATCCTCGGAA	rrs	Identification of *Candidatus* Coxiella massiliensis and other *Coxiella*-like bacteria in samples
Reverse: GCAACTAAGGACGAGGGTTG		
Probe: 6FAM-CAGCTCGTGTCGTGAGATGT-TAMRA		
Forward: 660f GGCGCICARATGGTTAARGA	groEL	Amplification of chaperonin genes for phylogenetic studies
Reverse: 1320r AACATCGCTTTACGACGA		

Technical Appendix Table 2. GenBank accession numbers of sequences identified during study of *Candidatus* Coxiella massiliensis infection

Accession no.	Tick host	Origin	Bacterial gene
***Candidatus* Coxiella massiliensis**			
KM079624	*Rhipicephalus sanguineus*	Laboratory colony in France, originally from Algeria	*rrs*
KM079625	*Rhipicephalus sanguineus*	Laboratory colony in France, originally from Algeria	*rrs*
KM079626	*Rhipicephalus turanicus*	Laboratory colony in France, originally from France	*rrs*
KM079627	*Rhipicephalus sanguineus*	Laboratory colony in France, originally from Algeria	*groEL*
KM079628	*Rhipicephalus bursa*	Laboratory colony in France, originally from France	*groEL*
**Other *Coxiella*-like bacteria from ticks**			
KM079617	*Amblyomma variegatum*	Senegal	*rrs*
KM079618	*Dermacentor silvarum*	France	*rrs*
KM079619	*Haemaphysalis concinna*	Far Eastern Russia	*rrs*
KM079620	*Haemaphysalis japonica douglasi*	Far Eastern Russia	*rrs*
KM079621	*Haemaphysalis japonica douglasi*	Far Eastern Russia	*rrs*
KM079623	*Rhipicephalus bursa*	Laboratory colony in France, originally from France	*rrs*

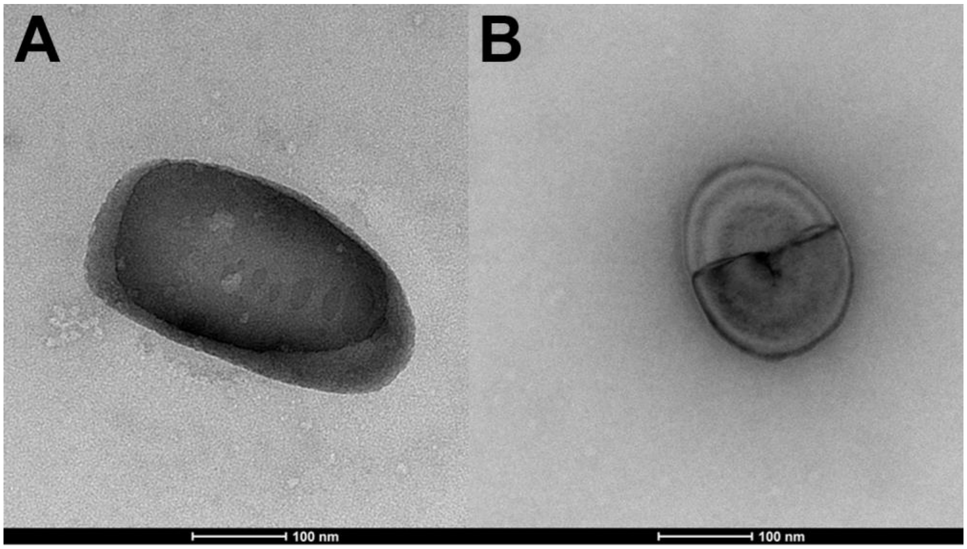

Technical Appendix Figure. Transmission electron micrograph of *Candidatus* Coxiella massiliensis taken with a Morgagni 268D microscope (Philips, Endhoven, the Netherlands) at an operating voltage of 60 kV. A) Ovoid form. B) Coccoid form.
